# 定位表达的新城疫病毒HN蛋白对荷瘤小鼠的抗肿瘤免疫作用

**DOI:** 10.3779/j.issn.1009-3419.2010.08.04

**Published:** 2010-08-20

**Authors:** 凯冰 王, 红 隋, 乐静 李, 曦 李, 磊 王

**Affiliations:** 1 150086 哈尔滨，哈尔滨医科大学附属第二临床医学院介入室 Interventional Department, the Second Hospital Affiliated to Harbin Medical University, Harbin 150086, China; 2 150040 哈尔滨，哈尔滨医科大学附属肿瘤医院内科 Department of Medicine, Tumor Hospital Affiliated to Harbin Medical University, Harbin 150040, China; 3 150001 哈尔滨，哈尔滨兽医研究所兽医生物技术国家重点实验室猪病组 Division of Swine Disease, National Key Laboratory of Veterinary Biotechnology, Harbin Veterinary Medicine, Harbin 150001, China

**Keywords:** HN蛋白, 细胞定位, 抗肿瘤免疫, 细胞毒性T淋巴细胞, Hemagglutinin-neuraminidase (HN) protein, Cellular localization, Anti-tumor immunity, Cytotoxic T lymphocyte

## Abstract

**背景与目的:**

新城疫病毒HN蛋白是新城疫病毒产生溶瘤作用的重要免疫原。在前期体外实验基础上，比较定位表达细胞不同部位的HN蛋白体内抗肿瘤免疫作用。

**方法:**

通过构建荷瘤小鼠，瘤内注射定位表达于细胞不同部位的新城疫病毒HN蛋白，即胞浆型（Cy-HN）、跨膜型（M-HN）、分泌型（Sc-HN）重组真核表达质粒，比较荷瘤小鼠的肿瘤生长速度，脾淋巴细胞增殖反应和细胞毒T细胞活性。

**结果:**

瘤内注射跨膜型重组真核表达质粒的荷瘤小鼠肿瘤生长缓慢，与瘤内注射胞浆型和分泌型重组真核表达质粒的荷瘤小鼠相比有统计学差异（第18天：*P*=0.022；第21天：*P* < 0.01），同时，该组荷瘤小鼠的淋巴细胞增殖反应和细胞毒T细胞活性也较高[M-HN *vs* Cy-HN, *P*=0.019; M-HN *vs* Sc-HN, *P*=0.043; M-HN *vs* pcDNA3.1(+), *P* < 0.01]。

**结论:**

定位表达于细胞不同部位的HN蛋白体内抗肿瘤免疫作用存在差异，跨膜型HN蛋白的重组DNA质粒能够提高荷瘤小鼠的特异性细胞免疫应答。

## Abstract (en)

**Background and objective:**

Hemagglutinin-neuraminidase (HN) protein of newcastle disease virus is an important immunogen for oncolysis. We designed three different expression plasmids encoding the HN protein targeted to different subcellular compartments: cytoplasmic (Cy-HN), secreted (Sc-HN) and membrane-anchored (M-HN). On the basis of antitumor effect *in vitro*, the aim of this study is to investigate the anti-tumor immunity effect of HN protein *in vivo*.

**Methods:**

In the present study, we developed a mouse model in order to evaluate the anti-tumor effect of the intratumorally injected modified HN proteins and the anti-tumor immunity by lymphocyte proliferative response and CTL activity test.

**Results:**

Although all three DNA constructs elicited an immune response, tumor-bearing mice intratumorally injected with M-HN demonstrated a significantly better anti-tumor effect than those injected with Cy-HN or Sc-HN (Day 18: *P*=0.022; Day 21: *P* < 0.01). It also showed that this anti-tumor effect was mediated by higher lymphocyte proliferative response and CTL activity in mice intratumorally injected with M-HN [M-HN *vs* Cy-HN, *P*=0.019; M-HN *vs* Sc-HN, *P*=0.043; M-HN *vs* pcDNA3.1(+), *P* < 0.01].

**Conclusion:**

The anti-tumor immunity of Newcastle disease virus HN protein is influenced by differential subcellular targeting. The membrane-anchored form of the HN protein appears to be an ideal candidate to improve the specific cellular immunity.

近年来，应用具有复制能力的溶瘤病毒治疗恶性肿瘤备受肿瘤学者的关注^[1-5]^。新近发现的新城疫病毒（newcastle disease virus, NDV）D90株具有体外抗肿瘤作用而对人正常组织细胞无毒性^[6, 7]^。新城疫病毒的这种选择性杀伤肿瘤细胞的特性的具体机制目前尚未阐明。随着对NDV抗肿瘤机制研究的不断深入，发现NDV的包膜糖蛋白血凝素–神经氨酸酶（hemagglutinin-neuraminidase, HN）是NDV抗肿瘤的主要功能性蛋白，是NDV抗肿瘤作用的主要分子基础^[8]^。

我们应用合理的生物学技术构建定位表达于细胞浆、细胞膜和细胞外的HN蛋白真核DNA质粒，即胞浆型（Cy-HN）、跨膜型（M-HN）和分泌型（Sc-HN）。前期工作中将构建的三种重组质粒体外转染肺癌A549细胞系，通过MTT和流式细胞仪方法检测证实三种重组质粒能够抑制肺癌A549细胞生长并存在差异，这种差异与跨膜型和分泌型HN蛋白的真核DNA质粒诱导A549细胞产生早期凋亡和晚期坏死的比率增加有关。电镜下和激光共聚焦显微镜下观察细胞内凋亡小体的存在^[9]^。基于体外实验的初步结论，本研究进一步研究胞浆型、跨膜型和分泌型HN蛋白的真核DNA质粒对荷瘤小鼠的抗肿瘤免疫作用。

## 1 材料与方法

### 1.1 细胞培养和重组真核表达质粒构建

人肺癌A549细胞系由哈尔滨兽医研究所国家重点实验室提供，常规培养（DMEM, Invitrogen, USA; 10%FBS, Invitrogen, USA）。以新城疫病毒D90株全基因为模板，以pcDNA3.1(+)为真核表达载体，构建胞浆型、跨膜型和分泌型DNA质粒并保存于哈尔滨兽医研究所国家重点实验室^[10]^。

### 1.2 建立裸小鼠皮下人肺腺癌移植瘤动物模型及瘤内接种步骤

裸小鼠纯合子nu/nu BALB/c，雌性，6周龄-7周龄，体重16 g-18 g，由上海斯莱克实验动物有限公司提供，饲养于哈尔滨兽医研究所动物实验中心屏障环境。所涉及的动物实验遵循动物实验操作规程。收集3×10^6^个肺癌A549细胞于150 μL PBS溶液（pH7.4）中制成单细胞悬液，接种于裸鼠大腿外侧皮下。每间隔2天-3天测量实体瘤的正交直径。一旦荷瘤在任何经线上达到25 mm，随机分入5个治疗组中：Cy-HN、Sc-HN、M-HN、空载体质粒pcDNA3.1(+)和生理盐水。分别接受瘤内多点注射重组质粒100 μg。每间隔5天（即d1，d5，d10，d15）注射1次，共4次。所有治疗的实验动物在初次治疗后21天处死，除非裸鼠出现严重的体重减轻或恶液质。肿瘤体积参考公式: V=（肿瘤最大截面积）^3/2^ ^[11]^。

### 1.3 脾脏淋巴细胞增殖实验检测小鼠细胞免疫功能

无菌取脾脏，制成脾单个核细胞悬液（V_脾细胞_:V_PBS_=1:4）。用0.75%Tris-NH_4_Cl（pH7.4）溶解红细胞。PBS洗涤3次，用Ficoll-isopaque solution提纯淋巴细胞制成单细胞悬液，RPMI-1640重悬并调整细胞密度（1×10^7^个/mL）。平铺于96孔平板中，100 μL/孔。每孔加入100 μL含有或不含有HN蛋白抗原（5 μg/mL），混匀。实验孔加入ConA（终浓度5 μg/mL）作为阳性对照，同时设无细胞孔作为调零孔。每孔设3个重复孔。用改良后的MTT比色法测定脾脏淋巴细胞增殖指数，即在37 ℃、5%CO_2_培养箱中培养72 h后，加入MTS（Promega, USA）20 μL继续培养5 h，培养结束前10 min内用酶标仪测定各孔A_570_值。淋巴细胞增殖指数（stimula- tion index, SI）=（加刺激细胞组OD值-混合淋巴细胞对照孔均OD值）/刺激细胞空白对照组OD值。

### 1.4 细胞毒性T淋巴细胞（cytotoxic T lymphocyte, CTL）实验

采用4 h ^51^Cr释放法^[12]^：①效应细胞：分离的脾淋巴细胞即为效应细胞CTL。②靶细胞：肺癌细胞A549重悬于不含碳酸氢钠的完全培养液。加入^51^Cr铬酸钠，37 ℃水浴中标记1 h，洗涤并调整细胞浓度为2×10^5^/mL。③体外致敏：实验组和对照组的淋巴细胞和经丝裂霉素C处理的A549细胞置37 ℃培养96 h。体外致敏的效应细胞经RPMI完全培养液洗涤，重悬。④细胞毒效应：向96孔U型板加入靶细胞，每孔100 μL（2×10^4^/孔）。分别按效应细胞:靶细胞=75:1、50:1和25:1向各孔加入100 μL不同效应细胞。每组均设3个复孔，阴性对照孔（自发释放）只加100 μL完全培养液，阳性对照孔（最大释放）加50 μL Zapoglobin（Beckman Coulter）细胞溶解剂。将96孔板置37 ℃培养4 h后，800 rpm离心培养板6 min，每孔吸取100 μL上清在液闪计数仪上测定每分钟放射活性（cpm值）。细胞杀伤活性按以下公式计算：CTL活性（%）=[（实验组cpm-自发释放cpm）/（最大释放cpm-自发释放cpm）]×100%。

### 1.5 数据分析

采用SPSS 15.0统计软件，所有变量均以Mean±SD表示。变量间差异比较用*One-way* *ANOVA*方差分析和*Fisher’s LSD*进行多重比较，*P* < 0.05为有统计学差异。

## 2 结果

### 2.1 细胞定位表达HN蛋白的重组DNA质粒对荷瘤小鼠肿瘤生长的影响

各种重组质粒对荷瘤小鼠肿瘤体积的变化见表 1。第9天，瘤内注射M-HN的小鼠肿瘤体积增长缓慢，同瘤内注射空载体pcDNA3.1(+)的相比有统计学差异。第12、15和18天，瘤内注射生理盐水组和空载体pcDNA3.1(+)组小鼠肿瘤体积同瘤内注射重组HN质粒组相比有统计学差异。第21天，各组荷瘤小鼠肿瘤体积增长出现明显差异，瘤内注射生理盐水组小鼠肿瘤体积最大，其次为空载体pcDNA3.1(+)组、Cy-HN、Sc-HN和M-HN（图 1）。比较第18天和21天荷瘤小鼠肿瘤体积的生长程度，瘤内注射M-HN的荷瘤小鼠肿瘤生长最慢（第18天：*P*=0.022;第21天：*P* < 0.01）。

**1:** 荷瘤小鼠经不同处理后肿瘤体积变化（*n*=8, Mean±SD） Variation of tumor size of A549 lung carcinoma tumor (*n*=8, Mean±SD)

Day^1^	Group 1: PC3.1 (+)	Group 2: Cy-HN	Group 3: Sc-HN	Group 4: M-HN	Group 5: Physiological saline	*P*^2^	*Post-hoc* test^3^
6	22.8±2.4 ^a^	20.9±3.6 ^a^	21.4±2.1 ^a^	20.7±1.6 ^a^	23.0±3.5 ^a^	0.336	NA
9	27.0±3.5 ^b^	23.9±1.6 ^b^	23.8±1.7 ^b^	22.4±0.8 ^b^	25.2±3.4 ^b^	0.007^*^	G1 < G4
12	32.4±0.2 ^c^	26.1±1.0 ^c^	26.6±2.0 ^c^	25.1±0.9 ^c^	31.8±0.3 ^c^	< 0.001^*^	G2=G3=G4 < G5 < G1
15	32.3±1.8 ^c^	27.3±0.5 ^c^	27.0±1.2 ^c^	25.9±2.1 ^c^	33.4±1.7 ^c^	< 0.001^*^	G2=G3=G4 < G1=G5
18	36.9±0.7 ^d^	31.8±0.8 ^d^	32.0±3.9 ^d^	29.4±1.4 ^d^	36.5±1.2 ^d^	< 0.001^*^	G4 < G2 < G3 < G1=G5
21	42.0±1.6 ^e^	34.0±0.8 ^e^	34.8±1.3 ^e^	32.7±0.3 ^e^	44.2±0.1 ^e^	< 0.001^*^	G4 < G2=G3 < G1 < G5
SD=standard deviation; Cy-HN: cytoplasmic HN; Sc: secreted HN; M: membrane-anchored HN; PC3.1(+): pcDNA3.1(+); NA=not available; ^1^ Linear mixed model was implemented to examine the differences between the time-points studied; ^2^ *One-way ANOVA* was performed; ^3^ *LSD* test or *Dunnett’s T3* test was used; ^*^ Significantly different among all groups, *P* < 0.05; Different letters meaned significant differences between various time-points, *P* < 0.05.							

**1:**
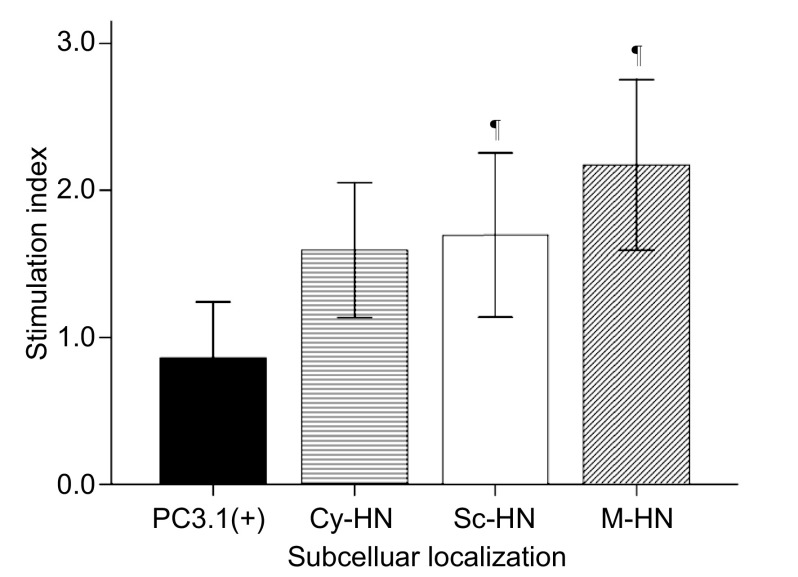
各组间淋巴细胞刺激指数比较。^¶^同PC3.1(+)组相比，*P* < 0.05。 Stimulation index in A549 cells transfected with PC3.1(+), Cy-HN, Sc-HN and M-HN. ^¶^ indicates significantly different from PC3.1(+), *P* < 0.05.

### 2.2 细胞定位表达HN蛋白的重组DNA质粒对脾淋巴细胞增殖的影响

瘤内注射M-HN的淋巴细胞刺激指数最高，同注射空质粒pcDNA3.1(+)组相比有统计学差异（2.18 *vs* 0.87, *P*=0.003）（图 1）。

### 2.3 CTL实验检测结果

跨膜型HN蛋白重组DNA质粒诱导的CTL活性最强，同其它各组相比有统计学差异[M-HN *vs* Cy-HN, *P*=0.019; M-HN *vs* Sc-HN, *P*=0.043; M-HN *vs* pcDNA3.1(+), *P* < 0.01]。胞浆型和分泌型HN蛋白重组DNA质粒诱导的CTL活性无统计学差异（*P*=0.656）（图 2）。

**2:**
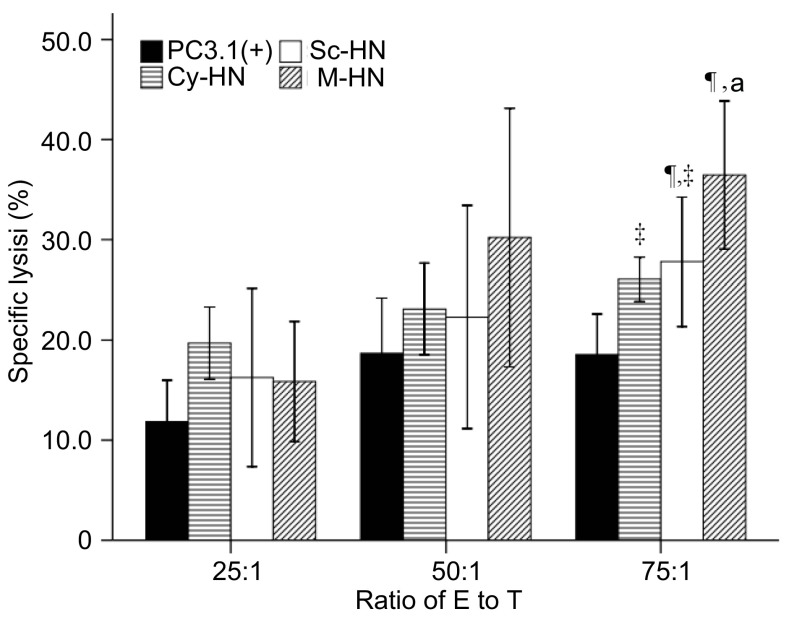
细胞毒性T淋巴细胞（CTL）实验。^¶^同PC3.1(+)组比较，*P* < 0.05;^‡^同M-HN组比较，*P* < 0.05，^a^同E/T比为25:1组，*P* < 0.05。 The CTL activity in tumor-bearing mice treated with PC3.1(+), Cy-HN, Sc-HN and M-HN. ^¶^ indicates significantly different from PC3.1(+), *P* < 0.05; ^‡^ denotes significantly different from M-HN, *P* < 0.05. ^a^ indicates significantly different from E/T ratio of 25:1, *P* < 0.05.

## 3 讨论

目前对新城疫病毒HN蛋白所介导的抗肿瘤机制尚未阐明，可能的机制包括：去除肿瘤细胞表面的唾液酸; 增强肿瘤细胞表面的粘附性和肿瘤细胞对淋巴细胞的共刺激作用; 促进效应细胞的活化和肿瘤细胞的凋亡。我们设想通过重组病毒结构调整其固有免疫效应来优化新城疫病毒所产生的溶瘤作用^[13, 14]^。将NDV的抗肿瘤*HN*基因进行克隆，以抗肿瘤基因的真核重组体的表达产物取代完整病毒，克服完整病毒所引起的副作用及可能带来的危险，从而具有非常可靠的安全性和细胞疫苗无法比拟的优越性。

本研究旨在探讨HN蛋白在宿主细胞不同部位表达的免疫原性的不同是否会带来肿瘤细胞识别和宿主细胞的细胞毒性作用的变化。体外实验的结果让我们看到通过重组病毒结构可以改善HN蛋白的抗肿瘤作用^[9]^。进一步研究通过构建荷瘤小鼠模型，比较3种重组质粒的抗肿瘤作用和免疫机制发现，瘤内注射跨膜型重组真核表达质粒的荷瘤小鼠肿瘤生长缓慢，与瘤内注射胞浆型和分泌型重组真核表达质粒的荷瘤小鼠相比有统计学差异（第18天：*P*=0.022;第21天：*P* < 0.01）。体外再刺激荷瘤小鼠脾淋巴细胞发现，其淋巴细胞刺激指数在瘤内注射跨膜型HN质粒组最高。应用标准的CTL实验检测定位表达HN蛋白的重组DNA质粒诱导的CTL效应，在效靶比（E/T）为75:1时，瘤内注射跨膜型质粒的裸鼠的CTL活性最强，同其它各组相比有统计学差异（*P* < 0.01），而胞浆型和分泌型HN蛋白的重组DNA质粒之间无统计学差异（*P* > 0.05）。

总之，我们的实验阐明了通过改变蛋白的细胞定位表达，HN蛋白的免疫原性可被显著提高。虽然本实验未能提供直接的质粒在体内的作用过程和细胞定位的证据，现有的数据亦不能直接如实反映质粒在荷瘤小鼠内的体内作用过程，但是我们的结果表明跨膜型HN蛋白的抗肿瘤免疫作用明显高于胞浆型和分泌型，其抗肿瘤作用机制有待进一步阐明。
